# *In silico* protein structure analysis of nine deleterious *NIT1* missense mutations identified in human cancers

**DOI:** 10.17912/micropub.biology.002210

**Published:** 2026-07-22

**Authors:** Anup Parajuli, Hung Phan, Susan Walsh

**Affiliations:** 1 Department of Molecular Genetics, The Ohio State University, Columbus, Ohio, USA; 2 Science and Mathematics Program, Soka University of America, Aliso Viejo, CA, USA; 3 Life Sciences Concentration, Soka University of America, Aliso Viejo, CA, USA

## Abstract

NIT1 is a tumor suppressor which functions as a metabolite repair enzyme to process deaminated glutathione (dGSH). Missense variants in NIT1 were analyzed from the COSMIC database to assess their structural and functional consequences. Of 59 missense variants identified, nine were flagged as deleterious. Homology modeling onto the *C. elegans* NitFhit structure revealed two mechanistically distinct classes: active site mutations predicted to disrupt the conserved Glu-Lys-Cys (EKC) catalytic triad and surface mutations predicted to perturb the Nit1-Fhit interaction interface. This work provides a structural basis for understanding NIT1 loss of function in human cancer.

**Figure 1. Structural and functional consequences of NIT1 missense variants were evaluated using multiple computational approaches:** 59 missense mutations were curated from COSMIC entry NIT1_ENST00000368008 for analysis. (A) Structural impact predictions were performed using MutationAssessor. Scores were classified into four functional impact categories based on the Functional Impact Score (FIS) thresholds: neutral (FIS < 0.8), low (0.8 < FIS > 1.93), medium (1.93 < FIS > 3.51), and high (FIS > 3.51). (B) Functional effects of amino acid substitutions were assessed with PROVEAN with scores > 0.5 considered damaging. (C) Domain architecture of the human NIT1 protein is shown to contextualize variant locations. An N-terminal mitochondrial targeting sequence (MTS) is indicated in blue, and the CN hydrolase homology domain is in yellow. Amino acids E86, K161, and C203 represent the catalytic triad (red). (D) A summary table lists the top nine missense variants predicted to be deleterious by MutationAssessor and PROVEAN. Mutations highlighted orange affect the active site, while mutations indicated in white affect the enzyme surface. (E) Structural superimposition of the human NIT1 homology model onto *Caenorhabditis elegans* Nit-Fragile Histidine Triad Fusion Protein (NitFhit; PDB: 1EMS, chain A), highlighting active site conservation. The human NIT1 model (teal) was generated using SWISS-MODEL based on the *C. elegans* NitFhit structure (tan). Active site residues from both structures are displayed as sticks colored by heteroatom. (F) Ribbon diagrams of two active site mutations onto the NIT1 homology model show the active site environment for the variants (K161R and E177K) with the mutant residue (blue) relative to the wildtype residue (tan) colored by heteroatom. (G) Surface representations demonstrate disruption of the predicted Fhit interaction surface. The wildtype surface (left) highlights the spatial positions of the original five key residues which were predicted to be deleterious (A50D, Q53H, T57M, N63K, and A74T) while the mutant surface displays all five mutations simultaneously superimposed onto a single structure, providing a composite view of their combined impact on the active site surface. All structures were visualized in UCSF ChimeraX.

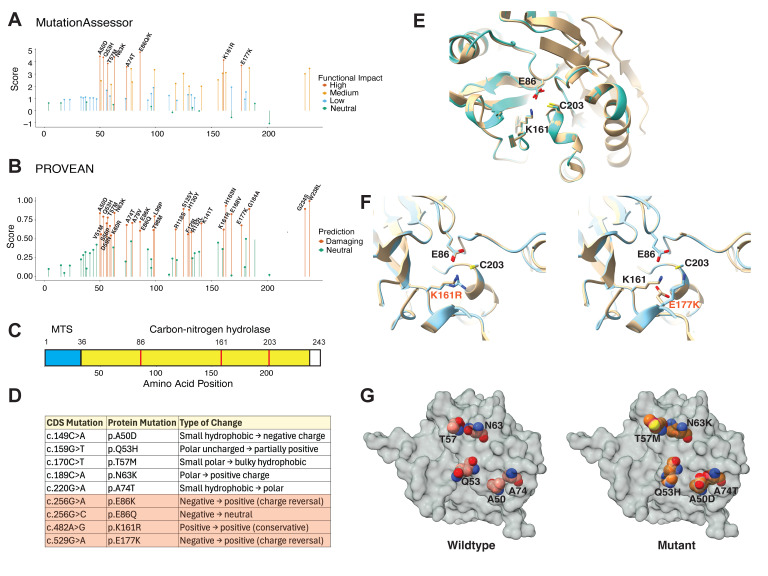



## Description

The Nit proteins belong to the nitrilase superfamily (Pace & Brenner, 2001). These proteins are broadly distributed across species and share conserved catalytic motifs but represent a distinct evolutionary lineage from more canonical nitrilases. In vertebrates, this subfamily includes NIT1 and NIT2. Rather than metabolizing nitriles, these two proteins function as amidases. Specifically, NIT1 hydrolyzes deaminated glutathione (dGSH) to α-ketoglutarate (α-KG) and cysteinylglycine (Peracchi et al., 2017; Van Schaftingen et al., 2026). In invertebrates, Nit1 is expressed as a single protein fused with the fragile histidine triad (Fhit) tumor suppressor protein, creating NitFhit (Pace et al., 2000; Semba et al., 2006; Pekarsky et al., 1998). However, in mammals, NIT1 and FHIT are encoded by distinct genes on different chromosomes. The human NIT1 forms tetramers and directly interacts with FHIT following the Rosetta Stone hypothesis (Mittag et al., 2023).

NIT1 is also a tumor suppressor. *NIT1* knockout mice displayed increased cellular proliferation, resistance to apoptosis, and higher incidence of chemically induced tumors (Semba et al., 2006). In colorectal cancer, NIT1 suppresses tumor proliferation through activation of the TGFβ1–Smad2/3 signaling pathway (Lin et al., 2018). Likewise, overexpression of *NIT1* in cell lines induces caspase-dependent apoptosis and reduces cell viability, paralleling Fhit’s effects on tumor suppression (Semba et al., 2006; Sun et al., 2009). Double knockout studies revealed that *FHIT ^–/–^ NIT1 ^–/–^* mice develop significantly more tumors than *FHIT* -deficient mice alone, suggesting that NIT1 and FHIT act in additive but distinct tumor suppressor pathways (Sun et al., 2009). Clinical data support this conclusion, with loss of *NIT1* expression observed in nearly half of human esophageal adenocarcinomas (Sun et al., 2009).

The dual role of NIT1 as both a metabolite repair enzyme and a tumor suppressor raises important questions about the molecular mechanisms by which it contributes to cancer. Unlike *FHIT* , which is frequently deleted due to its location at a fragile genomic site (Pekarsky et al., 1998), *NIT1* is less commonly inactivated by large homozygous deletions, as none were identified using the Co py N umber Variation An alysis Tool (CONAN) in the Catalogue of Somatic Mutations in Cancer (COSMIC) (Forbes et al., 2008; Forbes et al., 2017; Forer et al., 2010; Sondka et al., 2024). Of the fifty projects presented in The Cancer Genome Atlas Program’s Genomic Data Commons Data Portal, only two reported homozygous deletions in *NIT1* : CPTAC-3 (accession phs001287.v22.p7 ) and MP2PRT-ALL (accession phs002005.v1.p1 ) at frequencies of 0.13% and 0.07% (three cases total), respectively ( https://portal.gdc.cancer.gov/ ). In contrast, COSMIC lists numerous *NIT1* missense variants which were selected for this analysis, excluding nonsense mutations, UTR alterations, and promoter variants. Missense mutations are uniquely positioned to alter protein structure in ways produce subtle functional consequences, distinct from the binary loss of expression often associated with truncating or regulatory mutations. As such, these are the variants for which structural predictions are most informative and for which the resulting phenotype is most likely to reflect a partial or altered function rather than simple protein abolition. Here, we retrieved missense variant data from the COSMIC database, applied computational damage prediction tools to prioritize the most functionally deleterious substitutions, and modeled the resulting protein structures to assess their potential impact on NIT1 function ( **Figure 1** ).

COSMIC returned four transcript-level entries for *NIT1* ; we selected the primary entry (NIT1_ENST00000368008, UniProt entry Q86X76-5), which contained the largest number of catalogued mutations, as the basis for all subsequent analyses. This entry detailed 347 unique mutation types across 53,747 tested samples. Of these, 257 mutations fell outside the protein-coding region and were excluded, as they primarily affect gene expression or copy number rather than protein structure. The remaining 90 mutations mapped to the coding sequence, of which 59 were missense variants and carried forward for structural and functional analysis. These mutations were found across different tissue types, indicating no direct correlation between mutation and tissue type according to COSMIC. To evaluate the functional relevance of the identified *NIT1* missense variants, we applied a set of complementary computational prediction tools. MutationAssessor ( **Figure 1A** ) scored variants based on their potential to disrupt protein structure and function, with higher scores indicating greater predicted impact (Reva et al., 2011; Reva et al., 2007). PROVEAN ( **Figure 1B** ) assessed the effect of amino acid changes on protein activity, identifying substitutions predicted to impair function (Choi and Chan, 2015). CHASMplus was also used to look for driver mutations (Tokheim and Karchin, 2019) but failed to identify any of the *NIT1* missense mutations as statistically likely to be driver mutations (p values > 0.05). This is not surprising, given that *NIT1* is not classified as a driver gene by IntOGen (Gonzalez-Perez et al., 2013). By combining these approaches, we observed that mutations were distributed throughout the protein rather than localized to specific domains ( **Figure 1C** ). All nine variants flagged as high impact by MutationAssessor were also identified as damaging by PROVEAN, highlighting them as high-priority candidates for further functional characterization ( **Figure 1D** ).

To our knowledge, no experimental structure of human NIT1 exists. Therefore, to explore how these nine *NIT1* mutations might directly influence protein structure and therefore function, we generated our own human NIT1 model using SWISS-MODEL (Schwede et al., 2003) by aligning it with the experimentally determined X-ray structure of the *C. elegans* NitFhit homolog (PDB ID: 1EMS) (Pace et al., 2000). Importantly, only amino acids 37 to 243 were aligned because the human NIT1 sequence we referenced contains a N-terminal mitochondrial targeting sequence (Claros and Vincens 1996; Peracchi et al., 2017; Thumuluri et al., 2022), whereas the *C. elegans* structure does not (Pace et al., 2000). The two structures overlapped well, particularly the region containing the catalytic triad ( **Figure 1E** ). Analysis of this model identified the Glu-Lys-Cys (EKC) catalytic triad at residues E86, K161, and C203, consistent with the canonical nitrilase/amidase superfamily mechanism in which C203 acts as the nucleophilic thiol that attacks the carbonyl of dGSH to form an acyl-enzyme thioester intermediate, E86 acts as the general base for the thiol of C203, and K161 stabilizes the thioester intermediate through a hydrogen bond between its ε-amino group and the carbonyl of the thioester, with subsequent hydrolysis of the intermediate mediated by a water molecule positioned by E86 to release the product a-ketoglutarate (Pace et al., 2000; Pace & Brenner, 2001; Liu et al., 2013; Peracchi et al., 2017). Of the nine missense variants we studied, E86K, E86Q, K161R, and E177K map directly to or adjacent to this triad. E86K introduces a charge reversal that generates electrostatic repulsion with K161, predicted to displace it from its catalytic orientation, while E86Q eliminates the anchoring carboxylate entirely, abolishing the interaction that modulates K161's pKa without any steric perturbation. K161R substitutes the catalytic general acid with a bulkier guanidinium group whose substantially higher pKa renders it a poor proton donor under physiological conditions, with the structural overlay revealing an altered rotamer that further disrupts triad geometry **( Figure 1F )** . E177K, though not a triad residue, introduces a charge reversal from a negatively charged glutamate to a positively charged lysine, predicted to destabilize the active site **( Figure 1F )** .

The remaining five variants, A50D, Q53H, T57M, N63K, and A74T, cluster in the N-terminal region of the hydrolase domain distal from the catalytic triad **( Figure 1G )** , introducing charged or bulky residues into helical and loop segments that constitute the protein surface. In the *C. elegans* NitFhit structure, this region corresponds to the area encompassing the first β strand NS1 and first helix NH1 of the Nit domain, which form part of the solvent-exposed outer layer of the α–β–β–α sandwich fold (Pace et al., 2000). While NS13 is the strand most directly implicated in mediating physical contact with Fhit dimers, NS1 and NH1 contribute to the structural scaffold of the surface from which NS13 exits the globular core, and perturbations in this region could plausibly alter the overall surface topology in ways that indirectly affect the geometry of the NIT1-FHIT interaction. Given that the mammalian NIT1 and FHIT proteins interact directly (Mittag et al., 2023) and the sequence and structural homology between human NIT1 and the *C. elegans* template support conservation of surface topology in this region, the clustering of cancer-associated mutations here suggests they may compromise surface features relevant to FHIT interaction rather than catalysis directly. This interpretation is supported by data regarding the C203A mutation, which eliminates the nucleophilic cysteine in the catalytic triad yet does not abolish NIT1-mediated apoptosis or suppression of cyclin D1, demonstrating that NIT1's tumor suppressor function is separable from its hydrolase activity and likely depends on protein-protein interactions (Semba et al., 2006). Notably, mutations to C203 were not identified in any of the *NIT1* entries in the COSMIC database, which may reflect the fact that direct elimination of the catalytic cysteine may represent a lethal loss of function.

Collectively, our structural models indicate that these nine cancer-associated *NIT1* missense variants impair NIT1 function through two mechanistically distinct routes: disruption of the conserved catalytic triad or perturbation of the surface interface mediating tumor suppressor activity. These findings provide a structural basis for understanding how *NIT1* missense variants may contribute to cancer and highlight the need for experimental validation through biochemical characterization of both enzymatic activity and FHIT interaction.

## Methods

Somatic mutation data for *NIT1* (transcript ID: NIT1_ENST00000368008) were obtained from the COSMIC database (version 102; accessed September 29, 2025). The dataset was filtered to retain missense variants for downstream analysis. Functional impact prediction of the selected variants was performed using the OpenCRAVAT web server (version 2.13.0), with all variant coordinates referenced to the GRCh38/hg38 genome assembly (Pagel et al., 2020). This platform integrates multiple computational tools, including PROVEAN (Choi and Chan, 2015), CHASMplus (Tokheim and Karchin, 2019), MutationAssessor (Reva et al., 2011; Reva et al., 2007), and CONAN (Forer et al., 2010) to evaluate genes mutated in cancer. Mutant variants flagged by MutationAssessor and PROVEAN were designated as high-priority candidates for further characterization. Output files were exported in spreadsheet format and processed for visualization and analysis using R (4.5.2), with figures generated using the ggplot2 package (R core Team 2025; Wickham et al., 2019).

The mitochondrial targeting sequence (MTS) of human NIT1 was predicted using MitoProtII (v1.101) and DeepLoc-2.0, which collectively identified the N-terminal targeting region and informed the selection of residues 37–243 for structural analysis (Claros and Vincens 1996; Thumuluri et al., 2022). A three-dimensional homology model of human NIT1 was subsequently generated using the SWISS-MODEL server, employing the experimentally determined X-ray crystal structure of the *C. elegans* NitFhit homolog (PDB ID: 1EMS; resolution: 2.80 Å; sequence identity: 39.50%) as the modeling template. The resulting model exhibited a Global Model Quality Estimation (GMQE) score of 0.76 and a QMEANDisCo score of 0.73 ± 0.05, consistent with a reliable structural prediction based on moderate sequence homology. SWISS-MODEL builds the target structure by aligning the query sequence to the template scaffold, copying backbone coordinates, and refining side chains and loop regions through energy minimization. The human NIT1 homology model was then structurally aligned with chain A of 1EMS using the MatchMaker tool in UCSF ChimeraX, with superimposition restricted to residues 37–243 to exclude the N-terminal MTS absent in the *C. elegans* structure. The nine missense variants were mapped onto the human NIT1 homology model to assess their spatial distribution relative to the conserved active site and evaluate potential structural implications. All structural visualization and analysis were performed using UCSF ChimeraX (version 1.11.1) (Meng et al., 2023).
